# Inapropriate use of antibiotics effective against gram positive microorganisms despite restrictive antibiotic policies in ICUs: a prospective observational study

**DOI:** 10.1186/s12879-020-05005-7

**Published:** 2020-04-19

**Authors:** Hasan Selçuk Özger, Dolunay Merve Fakıoğlu, Kübra Erbay, Aslınur Albayrak, Kenan Hızel

**Affiliations:** 1 Faculty of Medicine, Department of Infectious Diseases, Gazi University, Ankara, Turkey; 2 Faculty of Pharmacy, Department of Clinical Pharmacy, Gazi University, Ankara, Turkey

**Keywords:** Antibiotic stewardship, Rational antibiotic use, Antibiotic resistance, Gram positive microorganisms, Inappropriate antibiotic use

## Abstract

**Background:**

Gram-positive spectrum antibiotics such as vancomycin, teicoplanin, daptomycin, and linezolid are frequently used in empirical treatment combinations in critically ill patients. Such inappropriate and unnecessary widespread use, leads to sub-optimal utilisation. However they are covered by the antibiotics restriction programme. This prospective observational study, evaluates gram-positive anti-bacterial utilisations in intensive care units (ICUs) with various evaluation criteria, to determine the frequency of inappropriate usage and the intervention targets required to ensure optimum use.

**Methods:**

This clinical study was conducted prospectively between 01.10.2018 and 01.10.2019 in the medical and surgical ICUs of Gazi University Faculty of Medicine Hospital, Turkey. The total bed capacity was 55. Patients older than 18 years and who were prescribed gram-positive spectrum antibiotics (vancomycin, teicoplanin, linezolid, and daptomycin) were included. Patients under this age or immunosuppressed patients (neutropenic,- HIV-infected patients with hematologic or solid organ malignancies) were not included in the study. During the study period, 200 treatments were evaluated in 169 patients. The demographic and clinical features of the patients were recorded. Besides observations by the clinical staff, the treatments were recorded and evaluated by two infectious diseases specialists and two clinical pharmacists at 24-h intervals from the first day to the last day of treatment.

SPSS software for Windows, (version 17, IBM, Armonk, NY) was used to analyse the data. Categorical variables were presented as number and percentage, and non-categorical variables were presented as mean ± standard deviation.

**Results:**

It was found that inappropriate gram-positive antibiotic use in ICUs was as high as 83% in terms of non-compliance with the selected quality parameters. Multivariate analysis was performed to evaluate the factors associated with inappropriate antibiotic use, increased creatinine levels were found to increase the risk of such use.

**Conclusions:**

In spite of the restricted antibiotics programme, inappropriate antibiotic use in ICUs is quite common. Thus, it is necessary to establish local guidelines in collaboration with different disciplines for the determination and follow-up of de-escalation of such use and optimal treatment doses.

## Background

Infection development is an important cause of morbidity and mortality in intensive care units (ICUs), leading to the widespread use of antibiotics [1]. It is reported that 41–85% of ICU patients use at least one antibiotic and antibiotic consumption is 10 times higher in ICUs compared to other units [2]. This widespread usage increases the unnecessary and inappropriate use of antibiotics and causes an increase in antimicrobial resistance [3]. Antimicrobial resistance typically increases the risk of poor clinical outcomes and death in ICUs patients [3]. Approximately 20–50% of hospitalized patients and 30–60% of patients in ICUs are prescribed unnecessary, inappropriate or sub-optimal antibiotic treatment [2–5]. Inappropriate use of empirical antibiotic treatments increases the frequency of early and late period mortality, length of hospital stay and healthcare-associated costs in ICU patients [4]. Antibiotic stewardship programmes are widely used to optimize antibiotic use in hospitals [6, 7]. Implementation of these programmes can lead to significant benefits in terms of clinical outcomes, reduced adverse events and lowered costs [3, 8]. To develop an effective program, it is necessary to determine the priority targets by evaluating inappropriate antibiotic use [6]. Antibiotic restriction lists should be implemented as part of Antimicrobial Stewardship Programmes (ASPs) and should be supported by other ASP strategies such as empirical treatment guidelines, de-escalation, preauthorisation and / or prospective audit and feedback [8]. As the effectiveness of antibiotic restriction programmes may decrease over time target-oriented revisions may be required to prevent the overuse or misuse of antibiotics [9].

Gram-positive spectrum antibiotics such as vancomycin, teicoplanin, daptomycin,and linezolid are frequently used in empirical treatment combinations in critically ill patients [10]. In Turkey, after beta-lactams and fluoroquinolones, these gram-positive antibiotics are reported to be the most commonly used antibiotics in ICUs [2]. Their widespread use, is thought to be not only inappropriate and unnecessary, but also responsible for sub-optimal utilisation. However, they are covered under the antibiotics restriction programme.

This prospective observational study, evaluates gram-positive anti-bacterial utilisations in ICUs with various evaluation criteria, the frequency of inappropriate usage and the intervention targets needed to ensure optimum use.

## Methods

This clinical study was conducted prospectively between 01.10.2018 and 01.10.2019 in the medical and surgical ICUs of Gazi University Faculty of Medicine Hospital, Turkey. The total bed capacity was 55. Between scheduled dates, all patients older than 18 years of age and using gram-positive spectrum antibiotics (vancomycin, teicoplanin, linezolid, and daptomycin) were included in the study. Recurrence use of gram-positive spectrum antibiotics in the same patients in different times were also included. Patients under the age of 18 years or immunosuppressed patients (neutropenic, and HIV-infected patients with hematologic or solid organ malignancies) were not included in the study.

The demographic and clinical features of the patients were recorded. Besides observations by the clinical staff, the treatments were recorded and evaluated by two infectious diseases specialists and two clinical pharmacists at 24-h intervals from the first day to the last day of treatment. Demographic data of patients (age, sex, body mass index, etc.), presence of comorbid diseases, Charslon comorbidity index, indications for antibiotic treatment, presence of sepsis or septic shock, clinical and laboratory findings (microbiological sampling results, creatinine clearance calculated using the Cockcroft-Gault equation, estimated glomerular filtration rate (eGFR) were recorded. This study was approved by the Institutional Review Board of Gazi University School of Medicine and was conducted according to the Declaration of Helsinki and Good Clinical Practice. (No: 02/14.01.2019).

### Definitions

The quality parameters evaluated for inappropriate antibiotic use in this study are given in Table 1.

**Table 1:** The Quality Parameters for Inappropriate use of antibiotics

Abbreviations	Criteria	Assessment Day	Non-Compliance Definition	References
**IUC-1**	**Antibiotic Treatment Indication** Doumented rationale for starting antibiotics in patients charts	1st day	No provide rationale of antibiotics	[9]
**IUC-2**	**Appropriate microbiological sampling** - At least 2 sets of blood culture -Culture from suspected infection site -Time for taking culture samples	1st day	Inadequate blood or suspected-infection site culture Collection of culture after antibiotic administration	[3, 10, 11]
**IUC-3**	**Antibiotic Dosage** -Antibiotic dose according to body weight -Loading dosage use^a^ -Adjustment of dosage according to the glomerular filtration rate (GFR)^b^	1st, 3st and 7st day	-Less than the recommended dose according to body weight or body mass index -No loading dose^a^ -No antibiotic dose adjustment according to GFR	[12]
**IUC-4**	**De-escalation**^c, d, e^ Discontinuation of antibiotic therapy based on microbiological results	3st and 7st day	Continuation of antibiotic therapy based on lack of antimicrobiological evidence	[3, 10, 11]
**IUC-5**	**Duration of treatment**^f^ Discontinuation of antibiotic therapy according to local or international guidelines	14st and 21^st^ day	-Longer treatment than recommended -Shorter treatment than recommended	[13–16]

*Abbreviations*: *IUC* Inappropriate use criteria

^a^Evaluated for vancomycin and teicoplanin

^b^Calculated with GFR cockroft formula

^c^The incubation time for samples other than blood cultures is 2 days and for blood samples a minimum of 5 days. For this reason, deescalation evaluation was performed on the 3rd and 7th days of treatment

^d^De-escalation assessment was only performed for empirical antibiotic treatment

eDe-escalation evaluation was not performed in patients whose treatment duration was less than 7 days

^f^De-escalation or withdrawal of the patient (discharge, transfer, death, etc.) has not been evaluated for treatment duration

### Inappropriate antibiotic use

Inappropriate antibiotic use is defined as non-compliance with at least one of the following quality parameters (documented antibiotic indication, appropriate microbiological sampling, appropriate dose, de-escalation and duration of treatment).

## Statistical method

SPSS software for Windows, (version 17, IBM, Armonk, NY) was used to analyse the data. Categorical variables were presented as number and percentage, and non-categorical variables were presented as mean ± standard deviation. The Chi-square test was used to compare the categorical variables. The fitness of the non-categorical variables to the normal distribution was evaluated with the Shapiro-Wilk test. The Mann-Whitney U test was used for the comparison of non-normally distributed variables variables respectively. In the univariate analysis, variables with a *p*-value of less than 0.20 and not correlated with each other were included in the logistic regression model. Charlson comorbidity index, use of a central catheter, treatment approach, C-reactive protein (CRP), sepsis, procalcitonin and creatinine levels were included in the logistic regression model. Values with a type-I error level of below 5% were considered as statistically significant.

## Results

During the study period, 200 treatments were evaluated in 169 patients. In 31 patients, gram-positive spectrum antibiotics were used more than ones. The clinical features of the patients were evaluated and are presented in Table 2.

**Table 2:** Clinical features of patients

Variables	N	%
Age, mean ± SD	63.9 ± 18.7	
Gender, Female	79	46,7
BMI (kg/m^2^),mean ± SD	26.5 ± 5.81	
Intensive Care Units (ICUs)		
Medical ICUs	95	56.2
Surgical ICUs	74	43.8
Duration of hospital stay (day), mean ± SD	16.4 ± 17.8	
Duration of ICU stay (day), mean ± SD	10.2 ± 14.4	
CCI, mean ± SD	4.40 ± 2.43	
Central venous catheters	100	59.2
Invasive mechanical ventilation	120	71
Renal failure		
CrCI, (mL/min) ≥ 50	89	52.7
CrCI, (mL/min) 30–49	25	14.8
CrCI, (mL/min) 10–29	45	26.6
CrCI, (mL/min) < 10	10	5.9
Intermittant renal replacement therapy	37	21.9
Continous renal relacement therapy	7	4.1

*Abbreviations*: *SD* standart deviation, *BMI* Body mass index, *ICU* Intensive care unit, *CCI* Charlson comorbidity index, *CrCI* Creatinine clearance

Regarding the use of antibiotics, the incidence of non-compliance with at least one of the determined quality parameters was 83%. The observed levels of non-compliance with the criteria of antibiotic indication, appropriate microbiological sampling, appropriate dosing, de-escalation and duration of treatment were; 47, 28, 26.5–35, 61.8–71.5 and 36%, respectively (Table 3).

**Table 3:** Frequency of inapropriate use of antibiotics (%)

	1. Day	3. Day	7. Day	14. Day	Total
IUC-1	47.0				
IUC-2	28.0				
IUC-3	26.5	35.0	35.0		
IUC-4		78.5	61.8		
IUC-5				36.0	
Total					83.0

*Abbreviations*: *IUC* Inappropriate use criteria

**Fig. 1:**
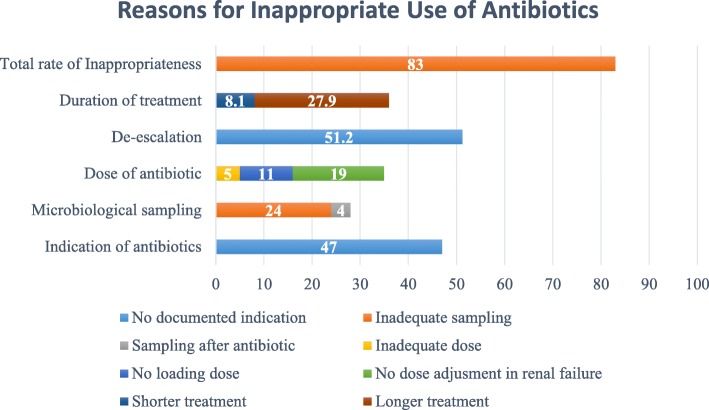
Reasons for inappropriate use of antibiotics

The determined quality criteria were also evaluated for non-compliance (Fig. 1). The most common inappropriateness for microbiological sampling was found to be associated with insufficient sampling (85.7%). Dosing errors were often associated with lack of dose adjustment according to renal clearance (54.3%). Longer duration of antibiotic use was the main reason for treatment duration inappropriateness 77.5 (%).

Factors associated with inappropriate use of antibiotics were analysed and are presented in Table 4.

**Table 4:** Factor associated with inappropriate use of antibiotics

	Inapropriate Use N(%)	Appropriate Use N(%)	*P* value
Age, mean ± SD^a^	64.3 ± 18.7	61.6 ± 20.8	.522
Gender^b^			
Female	78 (47)	16 (47.1)	.994
Male	88 (53)	18 (52.9)	
BMI (kg/m^2^), mean ± SD^a^	26.3 ± 6.24	27.0 ± 5.68	.228
CCI, mean ± SD^a^	4.51 ± 2.48	3.82 ± 2.35	.200
ICU^b^			
Medical	95 (57.2)	20 (58.8)	.864
Surgical	71 (42.8)	14 (41.2)	
Duration of Hospital Stay ± SD^a^ (Day)	23.2 ± 27.0	20.9 ± 26.6	.439
Duration of ICU stay ± SD^a^ (Day)	16.8 ± 25.8	16.5 ± 27.6	.591
Source of Infection^b^			
Sepsis	70 (49.3)	9 (29)	.040
Septic Schock	59 (30.5)	7 (20.6)	.091
Pneumoniae	111 (66.9)	20 (58.8)	.369
Blood Stream Infection (BSI)	46 (27.9)	5 (14.7)	.109
Others	20 (12.0)	11 (32.4)	.003
Unknown	32 (19.3)	3 (8.8)	.118
Antibiotic treatment approach^b^			
Emprical therapy	114 (68.7)	18 (52.9)	.078
Agent spesific therapy	52 (31.3)	16 (47.1)	
Central Venous Catheter	109 (65.7)	16 (47.1)	.041
Laboratory Paremeters^a^			
WBC (×10.e^3^/μL), mean ± SD	14.672 ± 19.179	14.802 ± 10.087	.460
PLT (×10.e^3^/μL), mean ± SD	221.879 ± 139.955	244.323 ± 156.829	.482
Lactate (mMol/L), mean ± SD	2.06 ± 1.70	2.22 ± 1.91	.644
GFR (mL/min), mean ± SD	50.6 ± 32.5	69.4 ± 26.8	.001
Cr (mg/ dL), mean ± SD	1.93 ± 1.74	0.90 ± 0.79	<.001
CRP (mg/L), mean ± SD	133 ± 92.8	156 ± 112	.041
Procalcitonin (ng/ mL), mean ± SD	18.7 ± 85.5	14.9 ± 68.5	.013

*Abbreviations*: *SD* standart deviation, *BMI* Body mass index, *CCI* Charslon comorbidity index, *ICU* Intensive care unit, *WBC* White blood cell, *PLT* Platelet, *GFR* Glomerular filtration rate, *Cr* Creatinine, *CRP* C-reactive protein

^a^Mann-Whitney U test was used

^b^Chi-squared test was used

Multivariate analysis was performed to evaluate the risk factors for inappropriate antibiotic use, and elevated creatinin levels ​​were found to increase this risk by approximately two times. (OR; 1.985, 95% CI 1.196–3.292, *p* = 0.008) (Table 5).

**Table 5:** Risk Factors for Inappropriate Antibiotic Use in Logistic Regression Analysis

	B	S.E	Sig.	O.R	% 95 C.I
CCI	−.042	.093	.650	.959	.799–1.150
Sepsis	−.552	.776	.477	.576	.126–2.635
Antibiotic tretament approach	−.504	.442	.255	.604	.254–1.438
Central venous catheter	.322	.415	.438	1.380	.612–3.112
CRP	−.003	.002	.136	.997	.993–1.001
Procalcitonin	.000	.002	.889	1.00	.995–1.005
Creatinine	.685	.258	.008	1.985	1.196–3.292

*Abbreviations*: *B* unstandardized regression weight, *CI* confidence interval, *OR* odds ratio, *SE* standard error, *CCI* Charslon comorbidity index, *CRP* C-reactive protein

## Discussion

As per the results of this study, inappropriate gram-positive spectrum antibiotics usage in ICUs was as high as 83%. Compliance with the evaluation of de-escalation was very low in the ICUs selected for this study. Renal failure increased the frequency of inappropriate antibiotic use by approximately 2-fold.

In Turkey, 71.3% of patients in ICUs are treated with antibiotics [2]. This widespread use is unnecessary and inappropriate. It is recommended that different quality parameters be used to evaluate inappropriate antibiotic usage. Dresser et al. advise considering uncertain indications, continuation of empirical treatment without evidence of infection, unnecessary prophylaxis, and drug contraindications as quality criteria for the evaluation of inappropriate antibiotic use [11].. For similar evaluations, Kallen et al. recommend considering appropriate microbiological sampling, therapeutic drug monitoring for vancomycin and aminoglycoside, surveillance cultures and periodic sharing of local resistance data [1].. The incidence of inappropriate empirical antibiotic use in ICUs reportedly varies between 14.1-and 78.9% due to differences in evaluation criteria [4, 12]. In Turkey, this incidence ranges from 30 to 50% [13–15].. The frequency of inappropriate antibiotic use as per our study is higher than that in the literature, since non-compliance with any of the criteria used in the study was considered sufficient to fulfil the definition of inappropriateness [6].

Since 2003, a national antibiotics restriction programme has been implemented in Turkey. Previous studies have shown that these programmes reduce the number of nosocomial infections, length of hospital stay, mortality and microbial resistance rates. The programme has had a positive effect on health expenditures [3, 16, 17]. However, several studies also showed that increased prescriptions of non-restricted antibiotics may eliminate these positive effects [2, 3]. The results of our study, show that the studied antibiotics, all of which are part of a restricted antibiotics programme, are used inappropriately and with high frequency. This indicates that inappropriate antibiotic use in ICUs cannot be prevented by restriction programmes alone and that the system should be supported by prospective audit and feedback mechanisms [8]. In fact the results of an intervention study conducted by Güçlü et al. was shown that antibiotic restriction programmes can be strengthened by supporting prospective control and feedback mechanisms [3].

The results of our study, revealed the continuation of antibiotics without microbiological evidence, as the most common factor adding to their inappropriate use. In ICUs, the de-escalation algorithm reduces the duration of treatment and frequency of microbial resistance without increasing mortality [3, 18–20]. Other studies conducted in Turkey, indicate such de-escalation is necessary in 10% of the cases [15]. On the other hand, the necessity of de-escalation in ICUs was shown to be higher. Mutters et al. reported that compliance with the evaluation of therapy discontinuation or de–escalation ranged from 2.4–8% [21]. In our study, the compliance in the early period of de-escalation (3 days) was found to be quite low. The frequency of de-escalation was slightly higher in the late period (5 days). Considering that the frequency of appropriate microbiological sampling is high, the above results may be attributed to late results (blood cultures) or late recognition. Despite the increased frequency compared to that in the early period, the frequency of late de-escalation was found to be low. The unwillingness of clinicians to discontinue treatment despite the results of the cultures is likely the important reason for this result. It appears that the restricted antibacterial programme alone does not seem to be sufficient for proper de-escalation in ICUs. Therefore it is crucial to develop an effective de-escalation strategy supported by local treatment guidelines.

Another important reason for inappropriate antibiotic usage in our study was the lack of proper antibiotic dose adjustment according to the eGFR. Renal failure and renal replacement therapies (RRTs), cause plasma concentration changes and affect drug concentrations [5]. RRTs, especially the continuous type, have also been shown to cause significant pharmacokinetic changes on the antibiotic groups that were evaluated in this work [22–24]. Therefore, antibiotic doses may remain suboptimal in ICU patients when compared to the normal population [5, 25, 26].. The frequency of RRTs in our study was 21.7%. Moreover, 6.7% of all patients received continuous RRT during the study. Also, elevated creatinine serum levels were found to be the major risk factor for the inappropriate use of antibiotics. Therefore, creatinine clearance changes need to be periodically evaluated to determine appropriate doses of antibiotics in collaboration with clinical pharmacists, infectious diseases specialists and clinical staff in ICUs [5].

There is study suffers from several limitations First, our data were collected from a single centre and the appropriateness of antibiotics was evaluated only for antibiotics effective against gram-positive microorganisms. These limitations prevent general assessments of the effectiveness of the national antibiotic restriction programme. Second, no global consensus currently exists on the criteria for evaluatng the inappropriate use of antibiotics in ICUs. Using different criteria may limit the applicability of the our study results. Third, this work did not evaluate the outcome measures related with the inappropriate use of antibiotics, such as mortality, duration of hospital or ICU stay, changing antimicrobial resistance patterns, and secondary infections (e.g. *C. difficile* infections). Despite all these limitations, this study successfully provided important insights into the appropriateness of antibiotic use towards improving the ASP. These results should be validated in the future via an interventional (before-after) study.

## Conclusion

In spite of the restricted antibiotic programme, inappropriate antibiotic use in ICUs is quite common. Appropriate use of antibiotics should be audited with predetermined quality parameters. In particular, it is necessary to establish local guidelines in collaboration with different disciplines for the determination and follow-up of de-escalation and optimal treatment doses. In patients undergoing RRT with increased risk of suboptimal concentration, antibacterial treatment doses should be individualized and closely monitored.

## Data Availability

All data generated or analysed during this study are included in this published article.

## Abbreviation

ASP: Antimicrobial Stewardship Programme
eGFR: estimated Glomerular Filtration Rate
HIV: Human Immunodeficiency Virus,
ICU: Intensive Care Unit
RRT: Renal Replacement Therapy
CRP: C-reactive protein
